# Retraction: LncRNA DANCR promotes migration and invasion through suppression of LncRNA-LET in gastric cancer cells

**DOI:** 10.1042/BSR-20171070_RET

**Published:** 2020-10-05

**Keywords:** DANCR, lncRNA-LET, EZH2, HDAC3

This Retraction follows an Expression of Concern relating to this article previously published by Portland Press.

This article is being retracted from *Bioscience Reports* at the request of the Editor in Chief and the Editorial Board following receipt of a notification from a reader, alerting the Editorial Board to duplication in the images used in Figures 1C, 1D, 1E, 1F, 2D and 2F.

The authors were contacted regarding the concerns raised and have not responded. Given the concerns with the Figures, the Editorial Board stands by the decision to retract.

